# Incremental prognostic value of stress phase entropy over standard PET myocardial perfusion imaging variables

**DOI:** 10.1007/s00259-023-06323-z

**Published:** 2023-07-10

**Authors:** Keiichiro Kuronuma, Robert J. H. Miller, Serge D. Van Kriekinge, Donghee Han, Ananya Singh, Heidi Gransar, Damini Dey, Daniel S. Berman, Piotr J. Slomka

**Affiliations:** 1 Departments of Medicine (Division of Artificial Intelligence in Medicine), Imaging, and Biomedical Sciences, Cedars-Sinai Medical Center, 8700 Beverly Blvd., Los Angeles, CA 90048 USA; 2 Department of Cardiology, Nihon University, Tokyo, Japan; 3 Department of Cardiac Sciences, University of Calgary, Calgary, AB Canada

**Keywords:** Myocardial perfusion imaging, Myocardial flow reserve, Positron emission tomography, Phase analysis, Phase entropy

## Abstract

**Purpose:**

Phase analysis can assess left ventricular dyssynchrony. The independent prognostic value of phase variables over positron emission tomography myocardial perfusion imaging (PET-MPI) variables including myocardial flow reserve (MFR) has not been studied. The aim of this study was to explore the prognostic value of phase variables for predicting mortality over standard PET-MPI variables.

**Methods:**

Consecutive patients who underwent pharmacological stress-rest ^82^Rb PET study were enrolled. All PET-MPI variables including phase variables (phase entropy, phase bandwidth, and phase standard deviation) were automatically obtained by QPET software (Cedars-Sinai, Los Angeles, CA). Cox proportional hazard analyses were used to assess associations with all-cause mortality (ACM).

**Results:**

In a total of 3963 patients (median age 71 years; 57% male), 923 patients (23%) died during a median follow-up of 5 years. Annualized mortality rates increased with stress phase entropy, with a 4.6-fold difference between the lowest and highest decile groups of entropy (2.6 vs. 12.0%/year). Abnormal stress phase entropy (optimal cutoff value, 43.8%) stratified ACM risk in patients with normal and impaired MFR (both *p* < 0.001). Among three phase variables, only stress phase entropy was significantly associated with ACM after the adjustment of standard clinical and PET-MPI variables including MFR and stress-rest change of phase variables, whether modeled as binary variables (adjusted hazard ratio, 1.44 for abnormal entropy [> 43.8%]; 95%CI, 1.18–1.75; *p* < 0.001) or continuous variables (adjusted hazard ratio, 1.05 per 5% increase; 95%CI, 1.01–1.10; *p* = 0.030). The addition of stress phase entropy to the standard PET-MPI variables significantly improved the discriminatory power for ACM prediction (*p* < 0.001), but the other phase variables did not (*p* > 0.1).

**Conclusion:**

Stress phase entropy is independently and incrementally associated with ACM beyond standard PET-MPI variables including MFR. Phase entropy can be obtained automatically and included in clinical reporting of PET-MPI studies to improve patient risk prediction.

**Supplementary Information:**

The online version contains supplementary material available at 10.1007/s00259-023-06323-z.

## Introduction

Left ventricular mechanical dyssynchrony is known to be associated with adverse events [1, 2]. Phase variables, a marker of the dyssynchrony, can be assessed by ECG-gated single photon emission computed tomography (SPECT) and positron emission tomography (PET) myocardial perfusion imaging (MPI) [3–5]. Our group recently reported that phase variables on SPECT-MPI were independently associated with adverse events after the adjustment of standard risk factors and SPECT-MPI variables [6]. Phase entropy on SPECT was shown to be the most promising variable to predict adverse events compared to other phase variables, phase bandwidth, and phase standard deviation (phase SD) [6].

A major advantage of PET-MPI is the ability to assess absolute myocardial blood flow (MBF) and myocardial blood flow reserve (MFR) in addition to conventional MPI variables such as myocardial perfusion, left ventricular volume, and ejection fraction [7]. It is well known that impaired MFR, a marker of coronary vascular dysfunction, is strongly associated with mortality and provides incremental prognostic information beyond conventional SPECT-MPI variables [7–9]. However, the prognostic value of stress phase variables after accounting for MFR and stress-rest change in phase variables has not been studied. The primary aim of the present study is to explore the independent and additive prognostic value of stress phase variables for predicting mortality beyond standard PET-MPI variables including MFR.

## Material and methods

### Study population

We enrolled 4735 consecutive patients referred for pharmacological rest-stress ^82^Rb PET-MPI at Cedars-Sinai Medical Center from January 2010 to December 2018. After excluding patients with early revascularization (within 90 days after the PET study; *n* = 418), ventricular paced rhythm (*n* = 263), and left bundle-branch block (*n* = 336), 3963 patients were included in the present study. The study complies with the Declaration of Helsinki and was approved by the institutional review board at Cedars-Sinai Medical Center. All participants gave informed consent.

### Clinical data

The patient demographic and clinical data including age, sex, body mass index (BMI), hypertension, dyslipidemia, diabetes, family history of coronary artery disease (CAD), smoking, history of peripheral vascular disease (PVD), right bundle-branch block (RBBB), and a history of prior CAD (previous myocardial infarction, percutaneous coronary intervention (PCI), and coronary artery bypass graft surgery (CABG)) [10] were collected on the day of the PET scan.

### Imaging acquisition

All patients underwent same-day, rest and pharmacological stress PET-MPI study using ^82^Rb with a Biograph 64 PET/CT scanner (Siemens Healthcare, Erlangen, Germany) or GE Discovery 710 (GE Healthcare, Waukesha, Wisconsin) scanner. A 6-min rest list-mode acquisition was started immediately before the injection of weight-based doses of 925–1850 MBq (25–50 mCi) of ^82^Rb. Pharmacologic stress was performed, and a 6-min stress imaging acquisition was simultaneously initiated with the start of the ^82^Rb administration. A low-dose helical CT was acquired prior to each rest and stress PET scanning for attenuation correction as previously described [8].

### MPI variable quantification

Myocardial perfusion and function variables, including rest and stress total perfusion deficit (TPD), LVEF, left ventricular end-diastolic volume (LVEDV), and left ventricular end-systolic volume (LVESV), were derived automatically using QPET software (Cedars-Sinai, Los Angeles, CA) [11]. Ischemic TPD (iTPD) was defined as stress TPD minus rest TPD. Abnormal MPI variables were defined as follows: iTPD ≥ 5%, LVEF < 45%, LVEDV > 120 mL, and LVESV > 70 mL [12, 13].

### Phase analysis

Three phase variables (entropy, bandwidth, and phase SD) were calculated automatically by QPET software (Cedars-Sinai, Los Angeles, CA). To obtain phase variables, count distributions were extracted and submitted to Fourier harmonic phase-angle analysis from gated left ventricular data [14]. The calculated phase distribution was represented on the histogram expressed in degrees from 0 to 360° corresponding to R-R interval. Histogram binning was performed using 60 6-degree bins. Phase variables were calculated from the phase histogram (Central Illustration). Briefly, bandwidth (expressed in degrees) is the smallest angle range that encompasses 95% of phase histogram measurements, phase SD (also expressed in degrees) is the SD of the histogram, and entropy (expressed in percentage) is the summation of (− fi × loge[fi]/loge[*n*]) for each bin (*i*), where fi is frequency in the *i*th bin, *n* is number of gating bins, and loge is the natural logarithm.

### MBF quantification

The 6-min list-mode data were reconstructed into 16 frames (12 × 10, 2 × 30, 1 × 60, and 1 × 120 s), and rest/stress MBF with a 1-tissue compartment kinetic model was calculated by the QPET software [15]. MBF and the spillover fraction from blood to myocardium were computed by numeric optimization. Stress and rest MBF values in mL/g/min were computed for each sample on the polar map. MFR was computed as the ratio of stress over rest MBF. Automated inter-frame motion correction was performed for all rest/stress MBF quantification [16]. Abnormal MFR was defined as equal or lower than 1.8 [17].

### Study endpoint

The primary endpoint was all-cause mortality (ACM). ACM was determined using internal hospital records as well as the Social Security Death Index, National Death Index, and California Non-Comprehensive Death File, which has previously been shown to be a reliable source for obtaining mortality status in the state of California [18].

### Statistical analysis

Categorical variables are shown as numbers and percentages, and continuous variables are shown as median values (IQR). Categorical variables were compared by the *χ*2 test, and continuous variables were compared by the Student *T* test or Mann–Whitney *U* test, as appropriate. Annual mortality rates were computed across deciles of entropy, quartiles of bandwidth, and deciles of phase SD. Since the bandwidth values are generated as multiple of 6, quartile was used. Relationships between all continuous variables are presented by the Spearman correlation coefficients. The optimal cutoff values of each phase variable to predict ACM was established by the Contal and O’Quigley method [19]. The Kaplan–Meier survival curves, stratified by global MFR (≤ 1.8) and the optimal cutoff of phase variables, were used to assess the primary outcome of ACM and compared using the log-rank test, followed by the Holm post hoc test [20].

A Cox regression model was used to assess associations between abnormal phase variables (based on the threshold defined by the Contal and O’Quigley method) and ACM. The following variables were included in multivariable model using a dichotomous variable: age, gender, body mass index, hypertension, dyslipidemia, diabetes, family history of CAD, smoking, PVD, prior history of CAD (prior history of MI, PCI, or CABG), RBBB, abnormal iTPD (≥ 5%), LVEDV (> 120 mL), LVESV (> 70 mL), LVEF (< 45%,), and MFR (≤ 1.8). The associations between phase variables including stress-rest change (Δphase variables) calculated as stress minus rest phase variables and ACM were also assessed as continuous variables. Due to multicollinearity, each phase variable was modeled in a separate model when continuous variables were used (Model 1 with phase entropy, Model 2 with phase bandwidth, and Model 3 with phase SD). Additionally, stress phase variables, rest phase variables, and Δphase variables were not modeled in the same model due to multicollinearity. The following variables were included in multivariable model when continuous variables were used: age (years), gender, body mass index (kg/m^2^), hypertension, dyslipidemia, diabetes, family history of CAD, smoking, PVD, prior history of CAD, RBBB, stress and rest TPD (%), LVEDV (mL), LVEF (%), MFR, and stress phase entropy and Δphase entropy (Model 1), stress phase bandwidth and Δphase bandwidth (Model 2), or stress phase SD and Δphase SD (Model 3). We checked the interactions in predicting ACM between phase entropy and age, male, iTPD, LVEDV, LVEF, and MFR. Since RBBB may affect the phase analysis, we repeated the analysis in patients without RBBB (*n* = 3657). To validate our results, the cohort of patients was randomly divided 1:1 into a derivation cohort and validation cohort as an internal validation. The optimal cutoff values of phase variables to predict ACM were derived from the derivation cohort and the analysis was repeated using those cutoff values in the validation cohort. Global *χ*^2^ analyses and likelihood ratios test were applied to evaluate the incremental fit of the model including each of the three phase variable compared with the model with standard PET-MPI variables (MFR, stress TPD, rest TPD, LVEDV, and LVEF) alone to predict ACM. All PET-MPI variables were modeled as continuous variables in global *χ*^2^ analyses. Reclassification analyses were conducted by calculating continuous net reclassification improvement (cNRI) and integrated discrimination improvement (IDI) at the median follow-up time of 5 years using the package “survIDINRI” in R [21]. cNRI is a measure to evaluate improvements in risk predictions, and it expresses the net percentages of patients with or without events correctly reclassified by the addition of a new marker. If an additional marker is associated with the outcome and additively predicts outcome, a significant (*p* < 0.05) and positive cNRI is expected [22]. A two-sided *p* < 0.05 was considered statistically significant. All statistical analyses were performed with R version 4.2.0 (R Foundation for Statistical Computing, Vienna, Austria) or STATA version 16 (StataCorp LP, College Station, TX).

## Results

### Patient characteristics and outcome

After exclusions, 3963 patients were included in the present study, and 923 (23.3%) patients died during median follow-up time of 5.2 [IQR, 3.2–7.3] years. Baseline patient characteristics are shown in Table 1. Patients who experienced ACM were older (median age, 76 vs. 69 years, *p* < 0.001) and more likely to have hypertension, diabetes, PVD, RBBB, and a history of CAD (Table 1).

**Table 1:** Patient characteristics

	Overall	ACM	No ACM	
	3963	923	3040	*p* value
Age, y	71.0 [63.0, 78.0]	76.0 [67.0, 84.0]	69.0 [62.0, 76.0]	< 0.001
Male sex	2267 (57.2)	541 (58.6)	1726 (56.8)	0.342
Body mass index, kg/m^2^	27.4 [24.1, 31.6]	25.8 [23.0, 29.9]	27.8 [24.5, 32.1]	< 0.001
Hypertension	3069 (77.4)	758 (82.1)	2311 (76.0)	< 0.001
Dyslipidemia	2659 (67.1)	572 (62.0)	2087 (68.7)	< 0.001
Diabetes	1307 (33.0)	373 (40.4)	934 (30.7)	< 0.001
Family history of CAD	634 (16.0)	89 (9.6)	545 (17.9)	< 0.001
Smoking	305 (7.7)	65 (7.0)	240 (7.9)	0.438
PVD	326 (8.2)	108 (11.7)	218 (7.2)	< 0.001
RBBB (%)	306 (7.7)	94 (10.2)	212 (7.0)	0.002
History of CAD	1272 (32.1)	388 (42.0)	884 (29.1)	< 0.001
History of MI	651 (16.4)	206 (22.3)	445 (14.6)	< 0.001
History of PCI	881 (22.2)	235 (25.5)	646 (21.2)	0.008
History of CABG	387 (9.8)	152 (16.5)	235 (7.7)	< 0.001

Values are shown as median [25th, 75th percentiles] or number (%) of patients. *ACM*, all-cause mortality; *CAD*, coronary artery disease; *CABG*, coronary artery bypass graft surgery; *MI*, myocardial infarction; *PCI*, percutaneous coronary intervention; *PVD*, peripheral vascular disease; *RBBB*, right bundle-branch block

### PET-MPI findings and outcomes

Patients with ACM had significantly higher stress and rest phase variables (phase entropy, phase bandwidth, and phase SD), ischemic TPD, LVEDV, and LVESV, and lower LVEF and MFR (Table 2). Phase variables at stress were lower than those at rest. The stress-rest change of phase variables was greater in patients without ACM than those with ACM (Table 2).

**Table 2:** Imaging characteristics

	Overall	ACM	No ACM	
	3963	923	3040	*p* value
Entropy				
Stress entropy, %	40.3 [34.0, 47.6]	44.5 [36.6, 53.5]	39.3 [33.3, 46.0]	< 0.001
Rest entropy, %	45.1 [38.6, 52.3]	47.0 [39.3, 55.7]	44.7 [38.4, 51.3]	< 0.001
ΔEntropy, %	− 4.1 [− 9.9, 1.3]	− 2.2 [− 7.6, 3.0]	− 4.8 [− 10.7, 0.7]	< 0.001
Abnormal stress entropy > 43.8%	1440 (36.3)	489 (53.0)	951 (31.3)	< 0.001
Bandwidth				
Stress bandwidth, °	36.0 [30.0, 54.0]	42.0 [30.0, 72.0]	36.0 [30.0, 48.0]	< 0.001
Rest bandwidth, °	42.0 [36.0, 66.0]	48.0 [36.0, 78.0]	42.0 [36.0, 60.0]	< 0.001
ΔBandwidth, °	− 6.0 [− 18.0, 6.0]	− 6.0 [− 18.0, 6.0]	− 6.0 [− 18.0, 0.0]	< 0.001
Abnormal stress bandwidth > 48°	999 (25.2)	368 (39.9)	631 (20.8)	< 0.001
Phase SD				
Stress phase SD, °	10.0 [6.9, 16.8]	12.9 [8.1, 23.3]	9.5 [6.7, 15.2]	< 0.001
Rest phase SD, °	13.5 [8.8, 21.9]	14.5 [8.9, 25.2]	13.2 [8.8, 21.2]	0.001
ΔPhase SD, °	− 2.2 [− 7.2, 1.1]	− 1.0 [− 5.4, 2.4]	− 2.6 [− 7.8, 0.7]	< 0.001
Abnormal stress phase SD > 13.5°	1353 (34.1)	449 (48.6)	904 (29.7)	< 0.001
Stress TPD, %	2.7 [1.0, 6.7]	4.6 [1.7, 11.0]	2.4 [0.9, 5.6]	< 0.001
Rest TPD, %	0.2 [0.0, 1.4]	0.6 [0.0, 4.1]	0.2 [0.0, 1.1]	< 0.001
Ischemic TPD, %	2.2 [0.9, 4.7]	3.3 [1.4, 6.1]	2.0 [0.8, 4.1]	< 0.001
Abnormal ischemic TPD ≥ 5%	909 (22.9)	320 (34.7)	589 (19.4)	< 0.001
LVEDV at rest, mL	81.3 [62.8, 106.5]	85.3 [62.3, 120.2]	80.5 [62.8, 103.4]	< 0.001
Abnormal LVEDV > 120 mL	685 (17.3)	233 (25.2)	452 (14.9)	< 0.001
LVESV at rest, mL	27.6 [17.6, 43.0]	33.3 [20.1, 57.6]	26.2 [17.0, 40.3]	< 0.001
Abnormal LVESV > 70 mL	416 (10.5)	174 (18.9)	242 (8.0)	< 0.001
LVEF at rest, %	66.4 [57.2, 73.1]	61.0 [48.5, 69.8]	67.4 [59.6, 73.9]	< 0.001
Abnormal LVEF < 45%	433 (10.9)	196 (21.2)	237 (7.8)	< 0.001
Stress MBF, mL/min/g	2.6 [2.0, 3.2]	2.2 [1.6, 2.9]	2.7 [2.1, 3.3]	< 0.001
Rest MBF, mL/min/g	1.1 [0.9, 1.4]	1.2 [0.9, 1.5]	1.1 [0.8, 1.4]	< 0.001
MFR	2.3 [1.8, 2.8]	1.8 [1.4, 2.3]	2.4 [1.9, 3.0]	< 0.001
Abnormal MFR ≤ 1.8	1043 (26.3)	453 (49.1)	590 (19.4)	< 0.001

Values are shown as median [25th, 75th percentiles] or number (%) of patients. Each Δphase variable was calculated as stress phase variable minus rest phase variable. *ACM*, all-cause mortality; *LVEDV*, left ventricular end-diastolic volume; *LVEF*, left ventricular ejection fraction; *LVESV*, left ventricular end-systolic volume; *MFR*, myocardial flow reserve; *SD*, standard deviation; *TPD*, total perfusion deficit

**Fig. 1:**
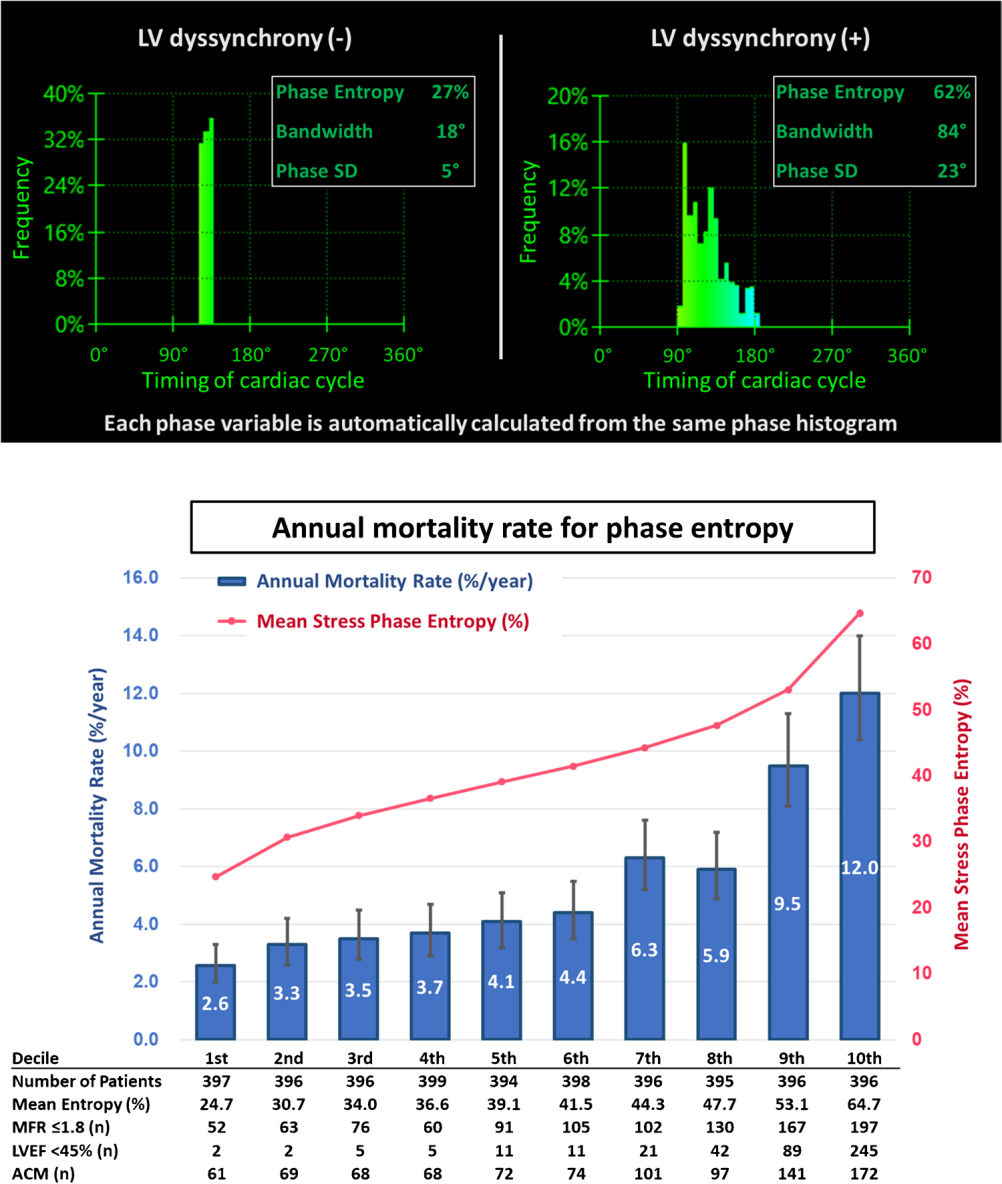
Top figure shows phase histograms of patients with and without dyssynchrony. The x axis represents the timing of one cardiac cycle in degrees (0 to 360° corresponds to the R-R interval). The y axis represents the frequency of end-systolic myocardium at a particular timing of the cardiac cycle. The frequency of myocardium contracting at each timing of the cardiac cycle. Bottom figure shows annualized mortality rate and deciles of stress phase entropy. The left y axis and blue bars show the annual mortality rates (%). The right y axis and pink line show mean post-stress phase entropy (%). ACM, all-cause mortality; LVEF, left ventricular ejection fraction; MFR, myocardial flow reserve; SD, standard deviation

Figure 1 shows annualized mortality rates across deciles of stress phase entropy. There was an increase in annualized mortality rates with increasing stress phase entropy. Patients in the 10th decile had 4.6-fold higher annualized mortality rate compared to those in the 1st decile (12.0 vs. 2.6%/year, *p* < 0.001). Similar results were observed for the other phase variables (Supplemental Figs. 1 and 2).

### Relationships between phase variables and other MPI variables

Supplemental Fig. 3 shows correlation coefficients between all continuous variables. There were strong positive correlations between three stress phase variables (*r* > 0.77). All phase variables negatively correlated with MFR and LVEF, and positively correlated with stress and rest TPD, LVEDV, and LVESV (Supplemental Fig. 3).

### The Kaplan–Meier analysis

**Fig. 2:**
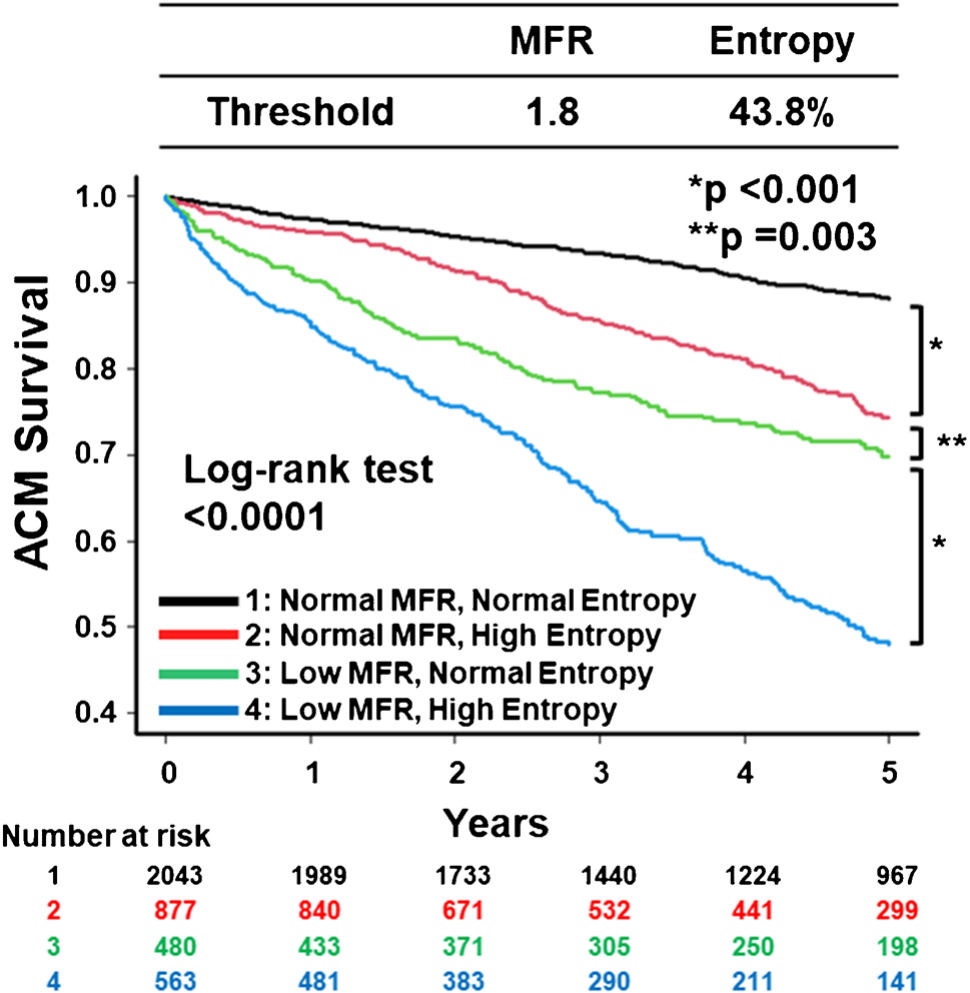
The Kaplan–Meier curves for ACM stratified by MFR and post-stress entropy. ACM, all-cause mortality; MFR, myocardial flow reserve

The optimal cutoff values of stress phase variables for mortality using the Youden index were 43.8% for phase entropy, 48° for phase bandwidth, and 13.5° for phase SD. The Kaplan–Meier survival curves for ACM were drawn according to the MFR abnormality and the optimal cutoff of phase entropy (Fig. 2). Combining phase entropy with MFR additively stratified the risk of ACM (Fig. 2). Similar results were observed using phase bandwidth and phase SD instead of phase entropy (Supplemental Fig. 4 and 5).

### Cox proportional hazards analysis

Table 3 shows the results of univariable and multivariable Cox regression analysis for prediction of ACM modeled as dichotomous variable. In the univariable analysis, all PET-MPI variables were associated with ACM (all *p* < 0.001) (Table 3). In the multivariable analysis, abnormal phase entropy (> 43.8%) was significantly associated with ACM (*p* < 0.001), but phase bandwidth and phase SD were not (both *p* > 0.1) (Table 3). Table 4 shows the results of stress phase variables and Δphase variables for prediction of ACM modeled as a continuous variable. In the univariable analysis, all stress phase variables and Δphase variables were associated with ACM (all *p* < 0.001) (Table 4). In the multivariable analysis, stress phase entropy was significantly associated with ACM, but Δphase variables and other stress phase variables were not (Table 4). The results of the final Cox model for each phase variable are shown in Supplemental Table 1–3. There was a significant interaction in predicting ACM between phase entropy and age (interaction *p* value = 0.049). In sub-analysis, the risk of ACM for patients with abnormal phase entropy was higher in young patients (< 65 years) than elderly patients (adjusted HR [95%CI], 2.11 [1.33–3.35]; *p* = 0.001 for young patients [< 65 years] and adjusted HR [95%CI], 1.27 [1.03–1.58]; *p* = 0.029 for elderly patients [≥ 65 years]). There was no interaction between phase entropy and male sex (*p* = 0.144), RBBB (*p* = 0.132), iTPD (*p* = 0.419), LVEDV (*p* = 0.425), LVEF (*p* = 0.329), and MFR (*p* = 0.087) (Supplemental Fig. 6). In the population excluding patients with RBBB (*n* = 3657), the cutoff values for predicting ACM were 43.8% for phase entropy, 42° for bandwidth, and 13.5° for phase SD. Abnormal phase entropy was significantly associated with ACM after the adjustment (adjusted HR [95%CI], 1.60 [1.27–2.02]; *p* < 0.001) (Supplemental Table 4).

**Table 3:** Unadjusted and adjusted HRs for ACM by dichotomous variables

	Univariable analysis		Multivariable analysis	
	Unadjusted HR (95%CI)	*p* value	Adjusted HR (95%CI)	*p* value
Age, y	**1.05 (1.04–1.05)**	** < 0.001**	**1.03 (1.03–1.04)**	** < 0.001**
Male sex	1.09 (0.96–1.25)	0.181	0.92 (0.80–1.06)	0.234
Body mass index, kg/m^2^	**0.95 (0.94–0.96)**	** < 0.001**	**0.96 (0.94–0.97)**	** < 0.001**
Hypertension	1.37 (1.16–1.62)	< 0.001	1.00 (0.84–1.20)	0.988
Dyslipidemia	**0.82 (0.72–0.94)**	**0.004**	**0.76 (0.66–0.88)**	** < 0.001**
Diabetes	**1.48 (1.30–1.69)**	** < 0.001**	**1.54 (1.34–1.77)**	** < 0.001**
Family history of CAD	0.58 (0.47–0.73)	< 0.001	0.80 (0.64–1.00)	0.054
Smoking	0.90 (0.70–1.16)	0.430	1.15 (0.89–1.49)	0.277
PVD	**1.82 (1.49–2.22)**	** < 0.001**	**1.33 (1.09–1.64)**	**0.006**
History of CAD	1.57 (1.38–1.79)	< 0.001	1.00 (0.86–1.17)	0.968
RBBB	1.46 (1.18–1.81)	< 0.001	1.13 (0.91–1.41)	0.260
Abnormal ischemic TPD, ≥ 5%	1.95 (1.70–2.23)	< 0.001	1.15 (0.98–1.35)	0.079
Abnormal LVEDV, > 120 mL	**1.87 (1.61–2.17)**	** < 0.001**	**1.48 (1.16–1.89)**	**0.002**
Abnormal LVESV, > 70 mL	2.36 (2.00–2.78)	< 0.001	0.92 (0.65–1.30)	0.622
Abnormal LVEF, < 45%	**2.62 (2.24–3.07)**	** < 0.001**	**1.38 (1.05–1.82)**	**0.021**
Abnormal MFR, ≤ 1.8	**3.23 (2.84–3.67)**	** < 0.001**	**1.99 (1.72–2.29)**	** < 0.001**
Abnormal stress entropy, > 43.8%	**2.52 (2.21–2.87)**	** < 0.001**	**1.44 (1.18–1.75)**	** < 0.001**
Abnormal stress bandwidth, > 48°	2.47 (2.17–2.82)	< 0.001	0.98 (0.77–1.25)	0.893
Abnormal stress phase SD, > 13.5°	2.36 (2.07–2.69)	< 0.001	1.19 (0.96–1.49)	0.120

Values in bold indicate significance (*p* < 0.05) after the adjustment. *ACM*, all-cause mortality; *CAD*, coronary artery disease; *HR*, hazard ratio; *LVEDV*, left ventricular end-diastolic volume; *LVEF*, left ventricular ejection fraction; *LVESV*, left ventricular end-systolic volume; *MFR*, myocardial flow reserve; *PVD*, peripheral vascular disease; *RBBB*, right bundle-branch block; *SD*, standard deviation; *TPD*, total perfusion deficit

**Table 4:** Unadjusted and adjusted HRs for ACM by continuous variables

	Unadjusted HR (95%CI)	*p* value	Adjusted HR (95%CI)	*p* value
Model 1				
Stress phase entropy, per 5%	**1.22 (1.19–1.25)**	** < 0.001**	**1.05 (1.01–1.10)**	**0.030**
ΔPhase entropy, per 5%	1.14 (1.10–1.18)	< 0.001	1.01 (0.97–1.06)	0.545
Model 2				
Stress phase bandwidth, per 5°	1.04 (1.04–1.05)	< 0.001	1.00 (0.99–1.01)	0.944
ΔPhase bandwidth, per 5°	1.02 (1.01–1.03)	< 0.001	1.01 (0.995–1.02)	0.309
Model 3				
Stress phase SD, per 5°	1.15 (1.13–1.18)	< 0.001	1.00 (0.96–1.03)	0.807
ΔPhase SD, per 5°	1.09 (1.05–1.13)	< 0.001	1.03 (0.998–1.07)	0.065

Values in bold indicate significance (*p* < 0.05) after the adjustment. Each Δphase variable was calculated as stress phase variable minus rest phase variable. *ACM*, all-cause mortality; *HR*, hazard ratio; *SD*, standard deviation

For the internal validation, we randomly divided all patients into a derivation cohort (*n* = 1981) and validation cohort (*n* = 1982). The optimal cutoff values derived from the derivation cohort to predict ACM were 44.0% for phase entropy, 48° for bandwidth, and 11.9° for phase SD. By using those values, only abnormal phase entropy (> 44.0%) among three phase variables was associated with ACM (adjusted HR [95%CI], 1.56 [1.17–2.09]; *p* = 0.003) (Supplemental Table 5).

### Incremental value of phase variables over conventional MPI variables and MFR

**Fig. 3:**
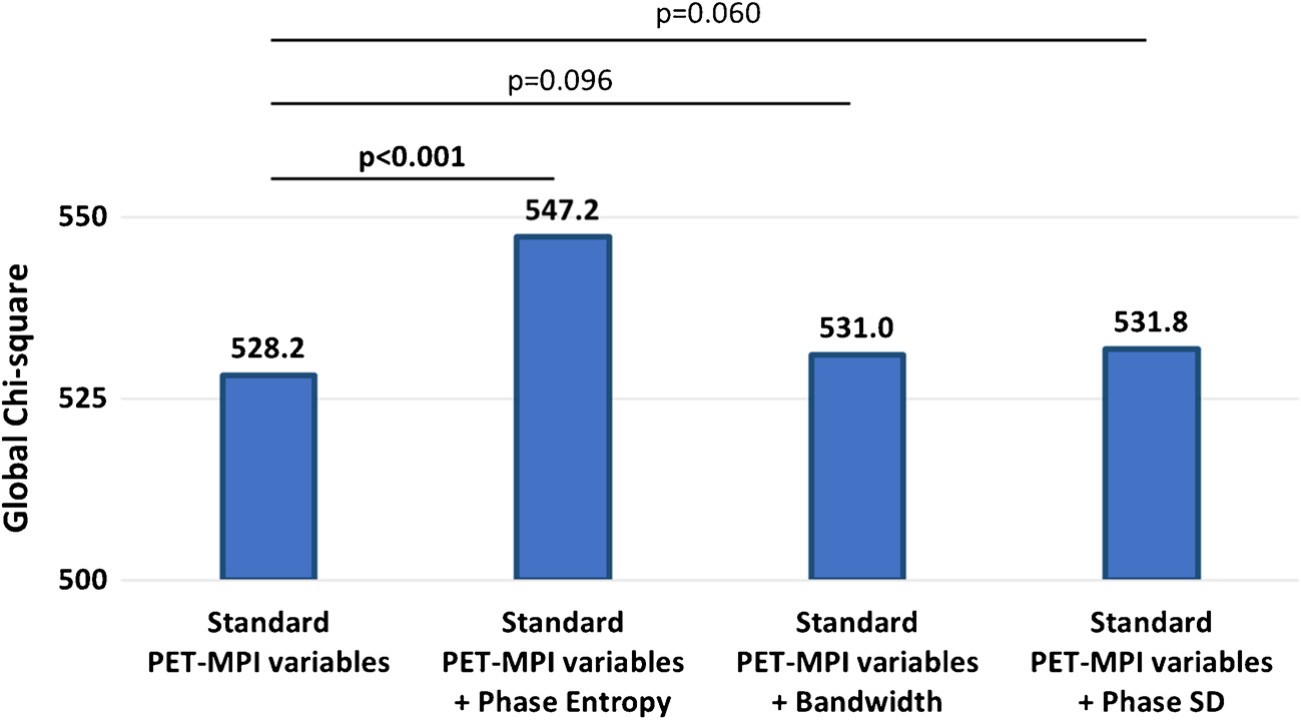
Improvement in model fit with post-stress phase variables for ACM prediction. Standard PET-MPI variables include stress and rest total perfusion defect, left ventricular end-diastolic volume, left ventricular ejection fraction, and myocardial flow reserve. All phase variables are stress values. ACM, all-cause mortality; PET-MPI, positron emission tomography myocardial perfusion imaging; SD, standard deviation

The global *χ*2 for the model adding stress phase entropy to standard PET-MPI variables (MFR, stress TPD, rest TPD, LVEDV, and LVEF) was significantly higher than that for standard PET-MPI alone (*p* < 0.001) (Fig. 3). When using phase bandwidth or phase SD instead of phase entropy, the global *χ*2 was not increased significantly (Fig. 3). Adding stress phase entropy significantly improved predictive performance for ACM compared to the model with standard PET-MPI variables alone (cNRI [95%CI], 0.127 [0.069–0.163]; *p* < 0.001 and IDI [95%CI], 0.008 [0.003–0.014]; *p* < 0.001), but the addition of phase bandwidth or phase SD did not (Table 5).

**Table 5:** NRI and IDI analysis for phase variables over standard PET-MPI variables in ACM prediction

	Continuous NRI (95%CI)	*p* value	IDI (95%CI)	*p* value
Standard PET-MPI variables alone	Baseline		Baseline	
Baseline + stress phase entropy	**0.127 (0.069 to 0.163)**	< 0.001*	**0.008 (0.003 to 0.014)**	< 0.001*
Baseline + stress phase bandwidth	0.051 (− 0.078 to 0.103)	0.252*	0.001 (− 0.001 to 0.006)	0.200*
Baseline + stress phase SD	0.080 (− 0.125 to 0.124)	0.120*	0.002 (− 0.001 to 0.005)	0.120*

Values in bold indicate significance (*p* < 0.05). Standard PET-MPI variables include stress and rest total perfusion defect, left ventricular end-diastolic volume, left ventricular ejection fraction, and myocardial flow reserve. *ACM*, all-cause mortality; *IDI*, integrated discrimination improvement; *NRI*, net reclassification improvement; *PET-MPI*, positron emission tomography myocardial perfusion imaging; *SD*, standard deviation. *Compared with baseline model (standard PET-MPI variables alone)

## Discussion

We investigated for the first time whether stress phase entropy, a measure of dyssynchrony of LV contraction, can be used to improve ACM prediction in patients undergoing PET-MPI. Compared to the studies of phase analysis using SPECT-MPI, little has been reported regarding PET-MPI, and none of the prior reports has studied incremental prognostic value of phase variables beyond standard PET-MPI variable [5]. In this study, we showed for the first time that stress phase entropy has independent prognostic value beyond standard PET-MPI variables including MFR. The main findings from this large observational study are as follows: (1) stress phase entropy was independently associated with ACM after the adjustment of standard PET-MPI variables including MFR, regardless if stress phase entropy was modeled as a dichotomous or continuous variable, (2) the delta changes of phase variables were not associated with ACM after the adjustment including stress phase variables.

Phase entropy, phase bandwidth, and phase SD are the principal phase analysis variables. Phase entropy is a mathematical expression to measure the overall disorder (or dispersion) of the phase histogram that is less likely to be influenced by outliers in the histogram [14, 23] or a moderate level of statistical noise. We recently showed that stress SPECT-MPI phase variables are independently associated with adverse cardiac events and that stress phase entropy was superior in prediction of events compared to stress phase bandwidth and stress phase SD [6]. In the present study, we found consistent results that annual morality rate increased with increasing phase variables (Fig. 1 and Supplemental Figs. 1 and 2). In multivariable Cox analysis including myocardial ischemia, LV size and function, and MFR, only phase entropy among the three phase variables was significantly associated with ACM. Adding phase entropy to those variables improved the discriminatory value and reclassification to predict ACM. Although there were mild to moderate correlations between phase variables and other MPI variables, standard PET-MPI variables do not consider dyssynchrony, or contraction heterogeneity related to left ventricular dysfunction.

We found that stress phase variables were lower than rest phase variables, indicating less dyssynchrony during pharmacologic stress, and patients without ACM had greater Δphase variables than those with ACM. The findings are consistent with previous studies using stress speckle-tracking echocardiography, in which dyssynchrony was improved after stress in patients without cardiac events or impaired coronary flow reserve and was comparable or worse after stress in those with cardiac events or impaired coronary flow reserve [24, 25]. However, our findings are different from those of previous studies using rest and stress phase variables with SPECT-MPI. Hida et al. showed that phase variables increased or dyssynchrony worsened after stress in overall population, and patients with multivessel disease had higher Δphase variables compared to those without multivessel disease [26]. AlJaroudi et al. showed that there was no significant difference in phase variables between stress and rest, even in patients with a large ischemia (reversible perfusion defect > 10%) [27]. The difference between our and the prior studies may be due to using different software and different stress protocol. The present study used QPET, and the other two studies used Emory Cardiac Toolbox [26, 27]. We explored the prognostic value of Δphase variables, and there was no significant association between Δphase variables and ACM after the adjustment including stress phase variables. Our findings suggest that stress phase entropy is the most promising variable among three phase variables including stress-rest change to predict ACM in patients undergoing PET-MPI studies. We confirmed the results through the internal validation analysis. In addition, there was a significant interaction between age and phase entropy (interaction *p* = 0.049) (Supplemental Fig. 6). Abnormal phase entropy may have a greater impact on ACM prediction in young patients than in elderly patients. Although phase entropy was moderately correlated with LVEDV and LVEF, there was no interaction in predicting ACM between phase entropy and LVEDV and LVEF (*p* > 0.05 for all interactions). The optimal cutoff values of stress phase entropy to predict ACM in the present PET population was higher than our recent study using SPECT (the cutoff value of phase entropy, 43.8 vs. 39.5%). This is likely because the population is older in the present study than the previous study (median age 71 vs. 64 years) and higher prevalence of coronary risk factors (e.g., hypertension, diabetes, and history of CAD). Those cutoff values were much lower than those for predicting response to cardiac resynchronization therapy (83° for bandwidth and 20° for phase SD) since the study was undertaken in a high-risk population (patients with heart failure, LVEF < 35%, and wide QRS) [28]. In addition, it has been shown that phase variables measured by different software are diverse and not interchangeable [29, 30]. Therefore, it is important to consider the patients’ clinical history and the type of phase analysis software to utilize phase variables in the clinical practice.

Since phase variables can be obtained fully automatically with high reproducibility [31], those can be readily incorporated into clinical PET-MPI reporting. In addition, phase entropy was consistently associated prognosis in patients undergoing SPECT and PET-MPI [6]. Therefore, of the three principal phase variables representing LV mechanical dyssynchrony on MPI, phase entropy would be the most promising variable for estimating a patient’s future risk.

### Study limitations

The present study has several limitations. This is a retrospective analysis of a single-center cohort data, and therefore, our results may not be generalized to other populations. We used ACM as an outcome and were not able to ascertain cardiovascular mortality in this retrospective study; however, reliability of identifying cause of death is limited [32]. We did not have detailed ECG information, including atrial ventricular conduction and arrhythmia such as premature ventricular or atrial contraction. Since all patients were included without exclusion, studies with gating errors may be included in this study. While the results from this large study appear to be robust regardless of the possibility of gating errors, it is important to note the importance of quality control, which may identify cardiac unreliable phase quantification. Although QRS duration on ECG is not available in this study, previous study has shown that phase variables had a stronger association with adverse cardiac events than QRS duration [33]. Finally, detailed medical treatment information at the time of the PET-MPI study and changes in the treatment after testing were not available in this study.

## Conclusion

Phase entropy has independent and incremental prognostic value for ACM over standard PET-MPI variables, including MFR. Phase entropy can be obtained automatically and routinely included in clinical reporting of PET-MPI studies to improve patient risk prediction.

### Supplementary Information

Below is the link to the electronic supplementary material.

Supplementary file1 (DOCX 719 KB)

## Data Availability

All data generated or analyzed during this study are included in this published article and the supplementary information files.
