# Supplementary material for: Incremental prognostic value of stress phase entropy over standard PET myocardial perfusion imaging variables
Source: Eur J Nucl Med Mol Imaging. 2023 Jul 10;50(12):3619–29. doi: 10.1007/s00259-023-06323-z (PMC10547643; doi:10.1007/s00259-023-06323-z)
Supplement: Supplementary file 1 — Supplementary file1 (DOCX 719 KB) [file 259_2023_6323_MOESM1_ESM.docx]

Supplemental Appendix

**Table of Contents**

[Supplemental Table 1. Adjusted HRs for ACM including phase entropy 2](#_Toc125379380)

[Supplemental Table 2. Adjusted HRs for ACM including phase bandwidth 3](#_Toc125379381)

[Supplemental Table 3. Adjusted HRs for ACM including phase SD 4](#_Toc125379382)

Supplemental Table 4. Adjusted HRs for ACM after excluding patients with RBBB (n=3657) …………………………………………………………………………………………………………………………………………………5

[Supplemental Table 5. Adjusted HRs for ACM in the validation cohort (n=1982) 6](#_Toc125379382)

[Supplemental Figure 1. Annualized mortality rate and deciles of stress bandwidth 7](#_Toc125379383)

[Supplemental Figure 2. Annualized mortality rate and deciles of stress phase SD 8](#_Toc125379384)

[Supplemental Figure 3. Correlation plots of continuous variables 9](#_Toc125379385)

[Supplemental Figure 4. Kaplan-Meier curves for ACM stratified by MFR and stress bandwidth 10](#_Toc125379386)

[Supplemental Figure 5. Kaplan-Meier curves for ACM stratified by MFR and stress phase SD11](#_Toc125379387)

Supplemental Figure 6. Forrest plots for the association between abnormal phase entropy and mortality according to baseline characteristics ……………………………………………………………………….[12](#_Toc125379385)

# Supplemental Table 1. Adjusted HRs for ACM including phase entropy

|  | Adjusted HR (95%CI) | p value |
| --- | --- | --- |
| Age, y | 1.03 (1.03-1.04) | <0.001 |
| Male sex | 0.86 (0.74-1.00) | 0.052 |
| Body mass index, kg/m^2^ | 0.96 (0.95-0.97) | <0.001 |
| Hypertension | 0.95 (0.79-1.13) | 0.551 |
| Dyslipidemia | 0.79 (0.69-0.91) | 0.001 |
| Diabetes | 1.43 (1.24-1.65) | <0.001 |
| Family history of CAD | 0.82 (0.66-1.02) | 0.076 |
| Smoking | 1.17 (0.90-1.51) | 0.237 |
| PVD | 1.29 (1.05-1.58) | 0.016 |
| History of CAD | 1.01 (0.87-1.18) | 0.889 |
| RBBB | 1.13 (0.91-1.41) | 0.275 |
| Stress TPD, % | 1.00 (0.99-1.02) | 0.918 |
| Rest TPD, % | 0.98 (0.96-1.01) | 0.137 |
| LVEDV, mL | 1.00 (1.00-1.00) | 0.997 |
| LVEF, % | 0.97 (0.97-0.98) | <0.001 |
| MFR | 0.57 (0.51-0.63) | <0.001 |
| Stress Phase Entropy, per 5% | 1.05 (1.01-1.10) | 0.030 |
| ΔPhase Entropy, per 5% | 1.01 (0.97-1.06) | 0.545 |

All MPI variables were modeled as a continuous variable. ACM, all-cause mortality; CAD, coronary artery disease; HR, hazard ratio; LVEDV indicates left ventricular end-diastolic volume; LVEF, left ventricular ejection fraction; MFR, myocardial flow reserve; PVD, peripheral vascular disease; RBBB, right bundle branch block; TPD, total perfusion deficit.

# Supplemental Table 2. Adjusted HRs for ACM including phase bandwidth

|  | Adjusted HR (95%CI) | p value |
| --- | --- | --- |
| Age, y | 1.03 (1.03-1.04) | <0.001 |
| Male sex | 0.84 (0.73-0.98) | 0.025 |
| Body mass index, kg/m^2^ | 0.96 (0.95-0.97) | <0.001 |
| Hypertension | 0.95 (0.80-1.14) | 0.593 |
| Dyslipidemia | 0.78 (0.68-0.90) | 0.001 |
| Diabetes | 1.42 (1.24-1.64) | <0.001 |
| Family history of CAD | 0.82 (0.65-1.02) | 0.073 |
| Smoking | 1.18 (0.91-1.52) | 0.221 |
| PVD | 1.29 (1.05-1.59) | 0.015 |
| History of CAD | 1.01 (0.87-1.18) | 0.861 |
| RBBB | 1.15 (0.92-1.43) | 0.210 |
| Stress TPD, % | 1.00 (0.99-1.02) | 0.701 |
| Rest TPD, % | 0.99 (0.96-1.01) | 0.169 |
| LVEDV, mL | 1.00 (1.00-1.00) | 0.863 |
| LVEF, % | 0.97 (0.96-0.98) | <0.001 |
| MFR | 0.56 (0.50-0.62) | <0.001 |
| Stress Phase Bandwidth, per 5° | 1.00 (0.99-1.01) | 0.944 |
| ΔPhase Bandwidth, per 5° | 1.01 (0.99-1.02) | 0.309 |

All MPI variables were modeled as a continuous variable. ACM, all-cause mortality; CAD, coronary artery disease; HR, hazard ratio; LVEDV indicates left ventricular end-diastolic volume; LVEF, left ventricular ejection fraction; MFR, myocardial flow reserve; PVD, peripheral vascular disease; RBBB, right bundle branch block; TPD, total perfusion deficit.

# Supplemental Table 3. Adjusted HRs for ACM including phase SD

|  | Adjusted HR (95%CI) | p value |
| --- | --- | --- |
| Age, y | 1.03 (1.03-1.04) | <0.001 |
| Male sex | 0.84 (0.73-0.98) | 0.024 |
| Body mass index, kg/m^2^ | 0.96 (0.95-0.97) | <0.001 |
| Hypertension | 0.96 (0.80-1.14) | 0.613 |
| Dyslipidemia | 0.79 (0.69-0.91) | 0.001 |
| Diabetes | 1.42 (1.23-1.64) | <0.001 |
| Family history of CAD | 0.82 (0.66-1.03) | 0.083 |
| Smoking | 1.17 (0.91-1.52) | 0.225 |
| PVD | 1.30 (1.06-1.59) | 0.014 |
| History of CAD | 1.01 (0.87-1.18) | 0.893 |
| RBBB | 1.15 (0.93-1.43) | 0.204 |
| Stress TPD, % | 1.00 (0.99-1.02) | 0.719 |
| Rest TPD, % | 0.99 (0.97-1.01) | 0.200 |
| LVEDV, mL | 1.00 (1.00-1.00) | 0.852 |
| LVEF, % | 0.97 (0.96-0.98) | <0.001 |
| MFR | 0.56 (0.50-0.63) | <0.001 |
| Stress Phase SD, per 5° | 1.00 (0.96-1.03) | 0.807 |
| ΔPhase SD, per 5° | 1.03 (0.99-1.07) | 0.065 |

All MPI variables were modeled as a continuous variable. ACM, all-cause mortality; CAD, coronary artery disease; HR, hazard ratio; LVEDV indicates left ventricular end-diastolic volume; LVEF, left ventricular ejection fraction; MFR, myocardial flow reserve; PVD, peripheral vascular disease; RBBB, right bundle branch block; SD, standard deviation; TPD, total perfusion deficit.

Supplemental Table 4. Adjusted HRs for ACM after excluding patients with RBBB (n=3657).

|  | Multivariable analysis | |
| --- | --- | --- |
|  | HR (95%CI) | p value |
| **Age, y** | **1.03 (1.03-1.04)** | **<0.001** |
| Male sex | 0.92 (0.79-1.07) | 0.272 |
| **Body mass index, kg/m2** | **0.96 (0.95-0.97)** | **<0.001** |
| Hypertension | 1.01 (0.84-1.22) | 0.896 |
| **Dyslipidemia** | **0.75 (0.65-0.87)** | **<0.001** |
| **Diabetes** | **1.56 (1.34-1.81)** | **<0.001** |
| **Family history of CAD** | **0.79 (0.62-1.00)** | **0.048** |
| Smoking | 1.15 (1.88-1.50) | 0.313 |
| **PVD** | **1.28 (1.02-1.59)** | **0.030** |
| History of CAD | 0.97 (0.83-1.14) | 0.711 |
| Abnormal Ischemic TPD, ≥5% | 1.12 (0.95-1.33) | 0.186 |
| **Abnormal LVEDV, >120mL** | **1.56 (1.20-1.84)** | **0.001** |
| Abnormal LVESV, >70mL | 0.91 (0.63-1.32) | 0.619 |
| Abnormal LVEF, <45% | 1.37 (1.02-1.84) | 0.036 |
| **Abnormal MFR, ≤1.8** | **2.06 (1.77-2.39)** | **<0.001** |
| **Abnormal Stress Entropy, >43.8%** | **1.60 (1.27-2.02)** | **<0.001** |
| Abnormal Stress Bandwidth, >42° | 0.88 (0.67-1.14) | 0.330 |
| Abnormal Stress Phase SD, >13.5° | 1.22 (0.97-1.52) | 0.087 |

Bold values indicate significance (p<0.05). ACM, all-cause mortality; CAD, coronary artery disease; HR, hazard ratio; LVEDV indicates left ventricular end-diastolic volume; LVEF, left ventricular ejection fraction; LVESV, left ventricular end-systolic volume; MFR, myocardial flow reserve; PVD, peripheral vascular disease; RBBB, right bundle branch block; SD, standard deviation; TPD, total perfusion deficit.

Supplemental Table 5. Adjusted HRs for ACM in the validation cohort (n=1982).

|  | Multivariable analysis | |
| --- | --- | --- |
|  | HR (95%CI) | p value |
| **Age, y** | **1.04 (1.03-1.05)** | **<0.001** |
| Male sex | 1.03 (0.84-1.25) | 0.807 |
| **Body mass index, kg/m2** | **0.95 (0.94-0.97)** | **<0.001** |
| Hypertension | 0.93 (0.72-1.19) | 0.539 |
| **Dyslipidemia** | **0.76 (0.62-0.92)** | **0.006** |
| **Diabetes** | **1.77 (1.45-2.16)** | **<0.001** |
| Family history of CAD | 0.79 (0.58-1.08) | 0.136 |
| Smoking | 1.13 (0.78-1.64) | 0.514 |
| **PVD** | **1.45 (1.10-1.90)** | **0.008** |
| History of CAD | 0.95 (0.77-1.17) | 0.630 |
| RBBB | 1.24 (0.91-1.69) | 0.174 |
| **Abnormal Ischemic TPD, ≥5%** | **1.25 (1.00-1.56)** | **0.047** |
| Abnormal LVEDV, >120mL | 1.21 (0.84-1.77) | 0.305 |
| Abnormal LVESV, >70mL | 1.29 (0.78-2.13) | 0.325 |
| Abnormal LVEF, <45% | 1.11 (0.75-1.62) | 0.612 |
| **Abnormal MFR, ≤1.8** | **1.96 (1.61-2.40)** | **<0.001** |
| **Abnormal Stress Entropy, >44.0%** | **1.56 (1.17-2.09)** | **0.003** |
| Abnormal Stress Bandwidth, >48° | 1.08 (0.79-1.48) | 0.642 |
| Abnormal Stress Phase SD, >11.9° | 0.91 (0.66-1.24) | 0.543 |

Bold values indicate significance (p<0.05). ACM, all-cause mortality; CAD, coronary artery disease; HR, hazard ratio; LVEDV indicates left ventricular end-diastolic volume; LVEF, left ventricular ejection fraction; LVESV, left ventricular end-systolic volume; MFR, myocardial flow reserve; PVD, peripheral vascular disease; RBBB, right bundle branch block; SD, standard deviation; TPD, total perfusion deficit.

# Supplemental Figure 1.


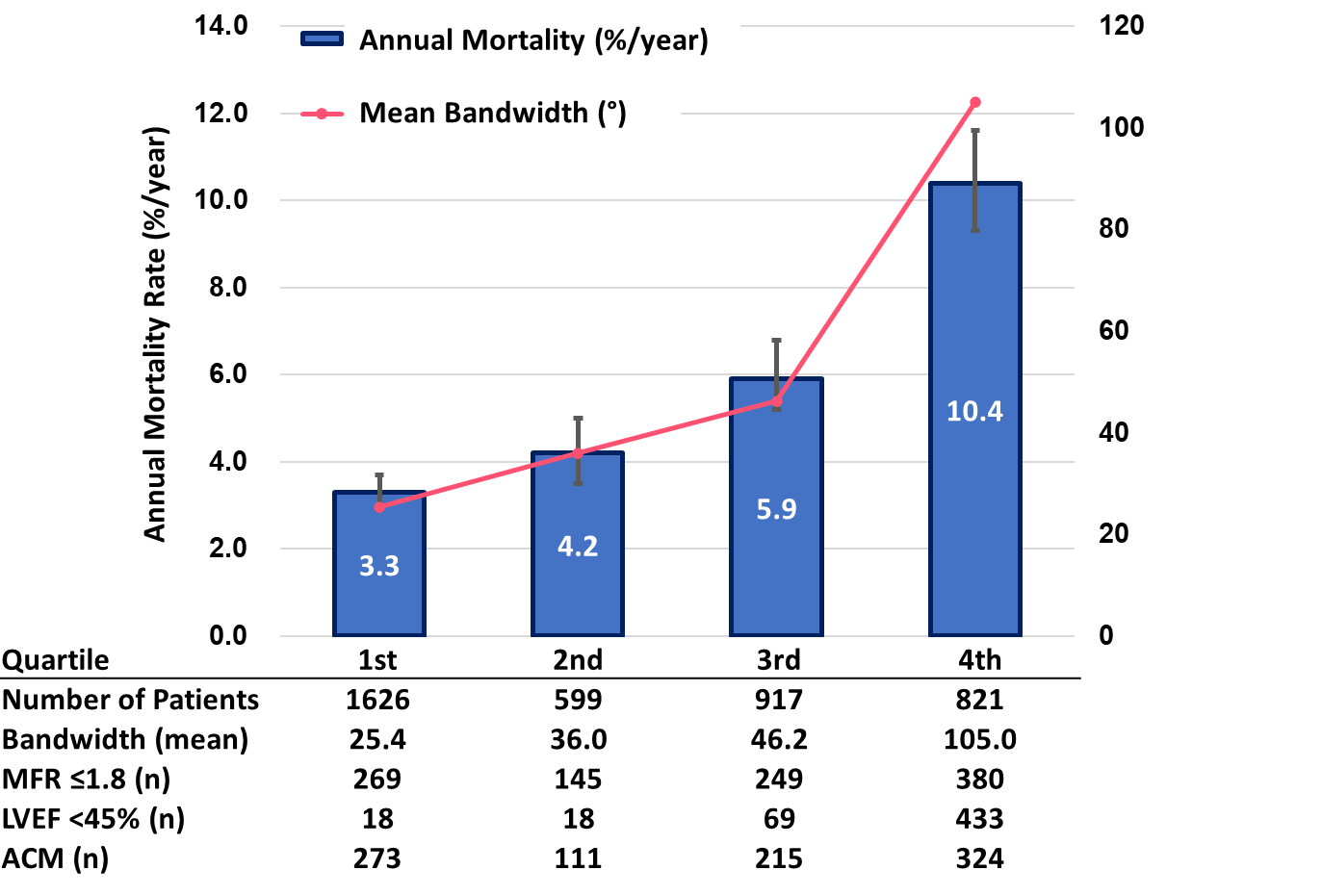


Annualized mortality rate and deciles of stress bandwidth. The left y axis and blue bars show the annual mortality rates (%/y). The right y axis and pink line show mean stress bandwidth (degree). ACM, all-cause mortality; LVEF, left ventricular ejection fraction; MFR, myocardial flow reserve.

# Supplemental Figure 2.


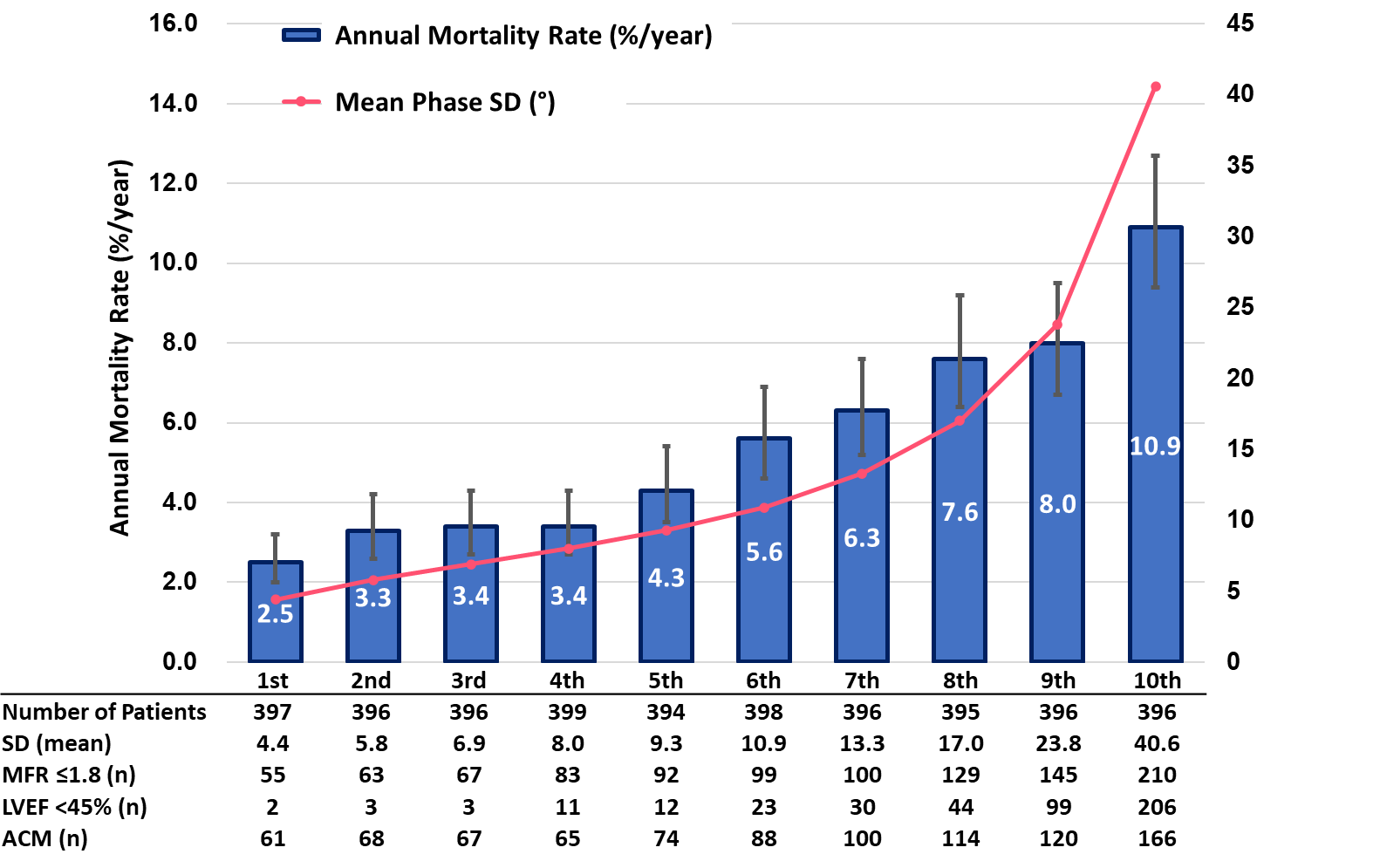


Annualized mortality rate and deciles of stress phase SD. The left y axis and blue bars show the annual mortality rates (%/y). The right y axis and pink line show mean stress phase SD (degree). ACM, all-cause mortality; LVEF, left ventricular ejection fraction; MFR, myocardial flow reserve; SD, standard deviation.

# Supplemental Figure 3.


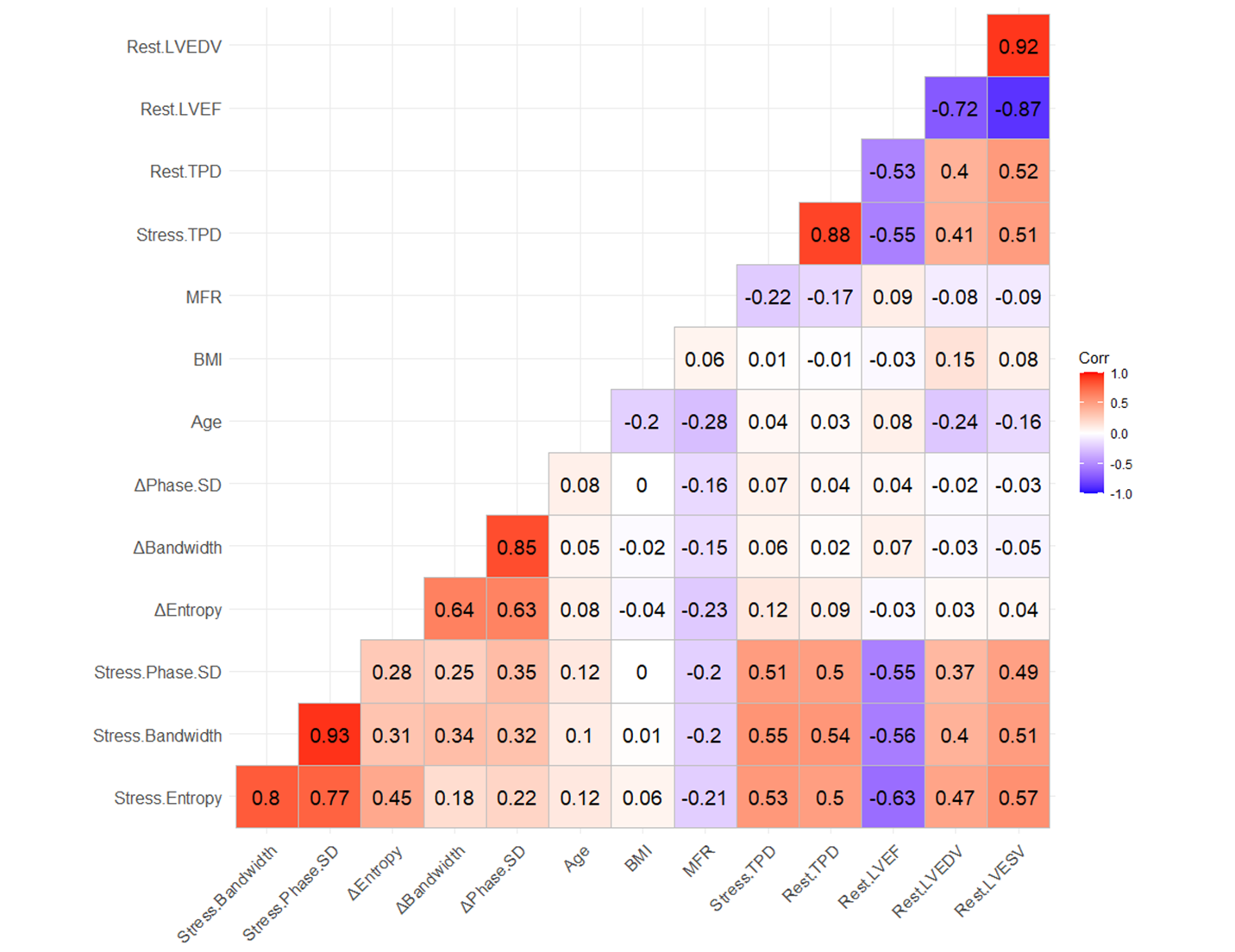


Correlation plots of continuous variables. BMI, body mass index; LVEDV indicates left ventricular end-diastolic volume; LVEF, left ventricular ejection fraction; MFR, myocardial flow reserve; SD, standard deviation; TPD, total perfusion deficit.

# Supplemental Figure 4.


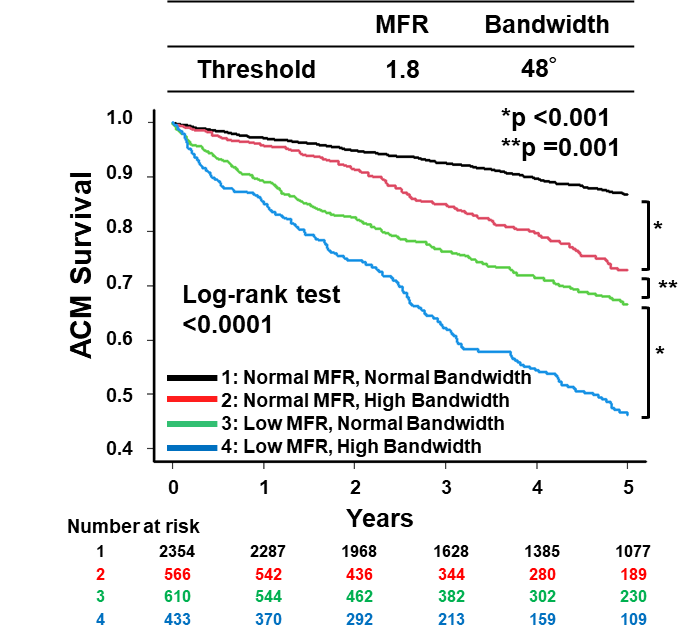


Kaplan-Meier curves for ACM stratified by MFR and stress bandwidth. ACM, all-cause mortality; MFR, myocardial flow reserve.

# Supplemental Figure 5.


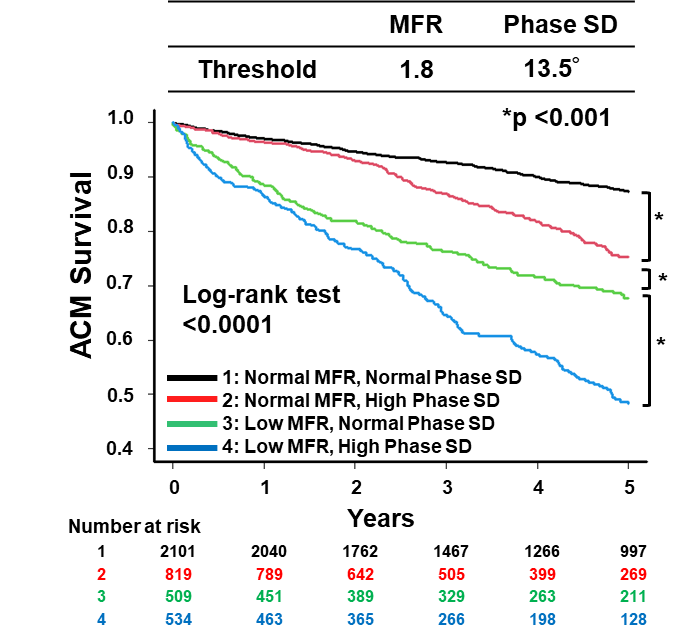


Kaplan-Meier curves for ACM stratified by MFR and stress phase SD. ACM, all-cause mortality; MFR, myocardial flow reserve; SD, standard deviation.

Supplemental Figure 6.


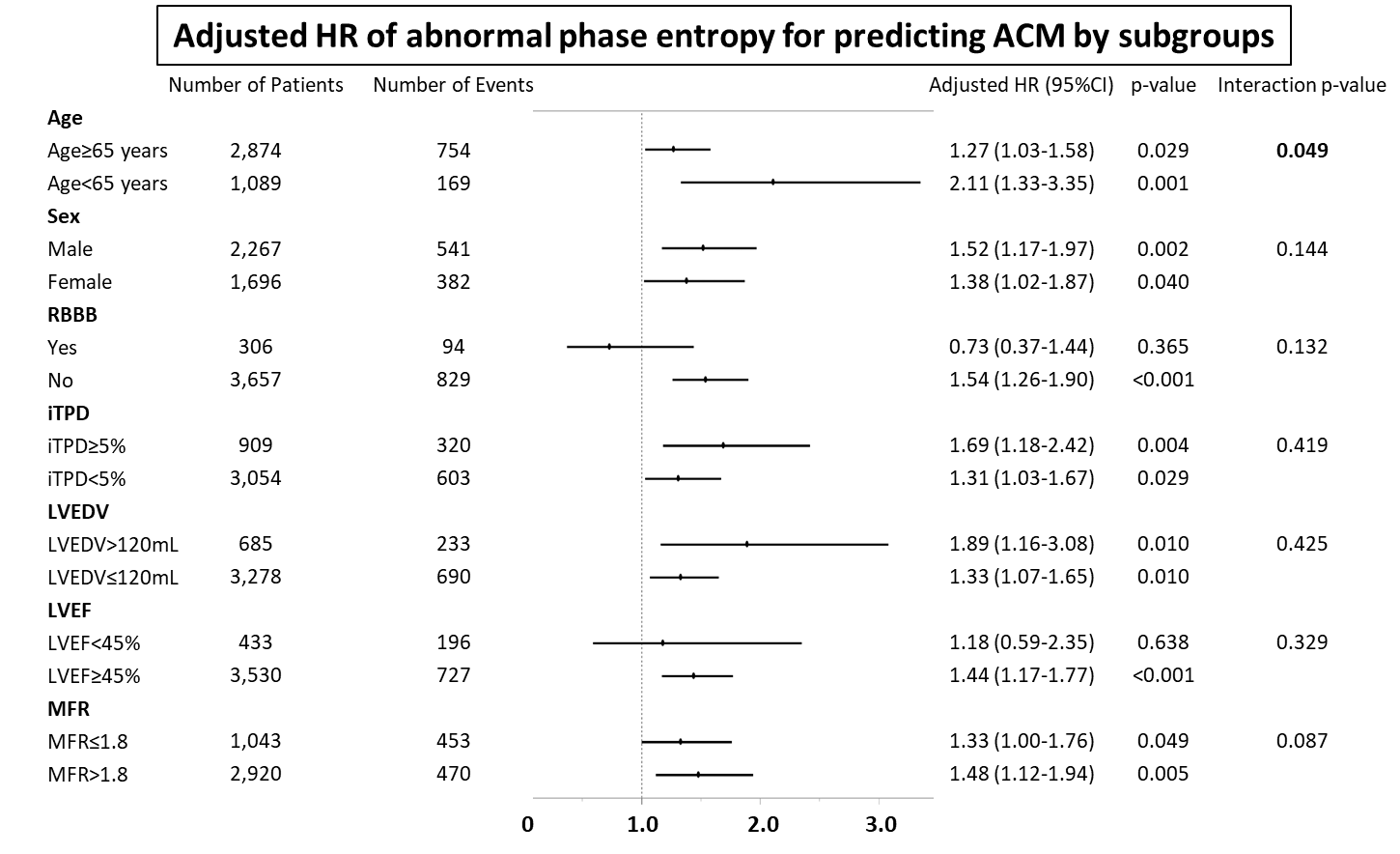


Forrest plots for the association between abnormal phase entropy and mortality according to baseline characteristics. iTPD, ischemic total perfusion deficit; LVEDV indicates left ventricular end-diastolic volume; LVEF, left ventricular ejection fraction; MFR, myocardial flow reserve; RBBB, right bundle branch block.
